# Molecular Mechanisms of Vitexin: An Update on Its Anti-Cancer Functions

**DOI:** 10.3390/ijms26125853

**Published:** 2025-06-18

**Authors:** Liyun Lu, Yinhua Deng, Junnan Li, Xing Feng, Hui Zou

**Affiliations:** 1 Key Laboratory of Study and Discovery of Small Targeted Molecules of Hunan Province, School of Pharmaceutical Sciences, Health Science Center, Hunan Normal University, Changsha 410013, China; 202320193569@hunnu.edu.cn (L.L.); dengyinhua244@hunnu.edu.cn (Y.D.); junnanli@hunnu.edu.cn (J.L.); 2 Key Laboratory of Model Animals and Stem Cell Biology in Hunan Province, Engineering Research Center of Reproduction and Translational Medicine of Hunan Province, Hunan Normal University, Changsha 410013, China; 3 Key Laboratory of Model Animals and Stem Cell Biology in Hunan Province, Manufacture-Based Learning and Research Demonstration Center for Human Reproductive Health New Technology of Hunan Normal University, Hunan Normal University, Changsha 410013, China

**Keywords:** vitexin, anti-cancer, molecular mechanism

## Abstract

Cancer remains a leading global health challenge, necessitating the exploration of novel therapeutic strategies. Vitexin (apigenin-8-C-β-D-glucopyranoside), a natural flavonoid glycoside with a molecular weight of 432.38 g/mol, is derived from plants such as mung bean, beetroot, and hawthorn. This compound features a distinctive C-glycosidic bond at the 8-position of its apigenin backbone, contributing to its enhanced metabolic stability compared to O-glycosidic flavonoids. Preclinical studies demonstrate that vitexin modulates critical cellular processes such as cell cycle progression, apoptosis, autophagy, metastasis, angiogenesis, epigenetic modifications, and tumor glycolysis inhibition. It exerts its effects by targeting key signaling pathways, including phosphoinositide 3-kinase/protein kinase B/mammalian target of rapamycin (PI3K/Akt/mTOR), nuclear factor kappa-light-chain-enhancer of activated B cells (NF-κB), and signal transducer and activator of transcription 3 (STAT3), and shows potential for combination therapies to enhance efficacy and overcome resistance. Advances in nanotechnology further enhance its bioavailability and delivery potential. This review comprehensively examines the current evidence on vitexin’s anticancer mechanisms, highlighting its multi-target therapeutic potential and future research directions.

## 1. Introduction

Cancer remains one of the most serious malignant diseases worldwide, threatening both physical and mental health due to its high morbidity and mortality rates. In recent decades, the global incidence of new cancer cases has increased significantly. Data from the International Agency for Research on Cancer (IARC) indicate that in 2022 alone, approximately 20 million new cancer cases were diagnosed, accompanied by nearly 10 million cancer-related deaths. Demographic projections suggest a dramatic escalation in this burden, with annual new cases predicted to reach 35 million by 2050, a 77% increase compared to 2022 levels [1]. These alarming statistics underscore the urgent need for innovative therapeutic strategies to combat this life-threatening disease.

Plants have served as a cornerstone of medicinal compounds for millennia, yet the molecular characterization of their bioactive constituents—particularly their anticancer potential—has only recently gained scientific attention [2]. Remarkably, over 60% of clinically approved chemotherapeutic agents trace their origins to natural products, underscoring the enduring value of botanical resources in drug discovery [3]. Plant-derived metabolites exhibit diverse therapeutic applications, with iconic examples including paclitaxel and the vinca alkaloids vinblastine and vincristine [4]. In recent years, flavonoids—a class of polyphenolic compounds—have emerged as promising candidates for anticancer therapy due to their multi-target mechanisms and favorable safety profiles. Notably, prunin, a citrus-derived flavonoid glycoside, has demonstrated potent anticancer activity by modulating the phosphatidylinositol 3-kinase/protein kinase B/mammalian target of rapamycin (PI3K/Akt/mTOR) and Wnt/β-catenin pathways in breast and colorectal cancers [5]. Other flavonoids, such as vitexin and kaempferol, also exhibit pleiotropic effects, including pro-apoptotic, anti-angiogenic, and antioxidant properties, positioning them as versatile agents for cancer management. This legacy highlights nature’s vast potential as a repository for novel lead compounds to address global health challenges.

In this review, we systematically analyze current evidence on the anticancer properties of vitexin, a naturally occurring flavonoid glycoside. We emphasize mechanistic insights into its effects across diverse cancer cell types, aiming to bridge existing knowledge gaps and inform future therapeutic development.

## 2. Sources, Chemistry, and Structural Activity Relationship of Vitexin

### 2.1. Botanical Sources and Pharmacological Properties

Vitexin (apigenin-8-C-β-D-glucopyranoside) is a naturally occurring flavonoid glycoside widely distributed in medicinal and edible plants, such as mung bean [6], beetroot [7], hawthorn [8], bamboo [9], Passiflora [10], and others. This compound exhibits a broad spectrum of pharmacological activities, including anticancer [11,12], antioxidant [13,14], anti-inflammatory [15,16], neuroprotective [17], and cardioprotective [18,19] effects. Additionally, vitexin has been reported to offer potential health benefits in areas such as nicotine cessation [20], hair regeneration [21], and nociception [22].

Analysis of the Web of Science Core Collection database (search query: TI = (vitexin)) reveals a significant increase in publications focusing on this compound, with an average annual output of 20.3 ± 2.8 papers prior to 2019, rising to 31.6 ± 4.5 papers between 2020 and 2023 (*p* < 0.01). This growing attention underscores its potential as a promising natural compound for therapeutic development. The following table provides a comprehensive overview of the plant sources of vitexin, highlighting its natural distribution and potential for extraction and utilization in biomedical research (Table 1).

### 2.2. Chemical Profile and Pharmacokinetic

**Figure 1:**
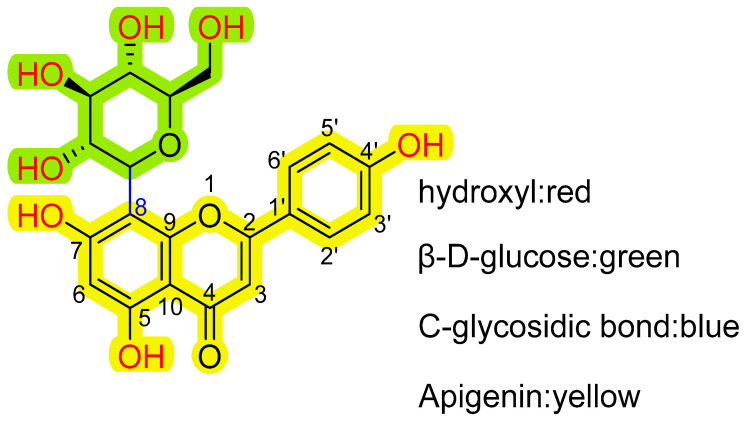
Chemical structure of vitexin.

Chemically, vitexin is known as 8-D-glucosyl-4′,5,7-trihydroxy-flavone, with the molecular formula C21H20O10 and a molecular weight of 432.38 g/mol. It is a relatively small polar molecule with a LogP value of 1.28 [35]. Structurally, vitexin consists of an apigenin backbone with a carboside group attached at position 8, as depicted in Figure 1. The addition of the carboside group significantly enhances its antioxidant and antitumor properties. When a hydroxyl group is located at positions 3 or 5 within the benzopyranone structure, the compound can chelate metal ions, potentially reducing its biological activity [36]. Vitexin contains seven hydroxyl groups, which may play a crucial role in its biological activity. Notably, the dihydroxyl structure in the A ring has been identified as an effective contributor to free-radical scavenging in flavonoids. The relative stability of hydroxyl radicals in vitexin has been ranked as 4′-OH > 7-OH > 5-OH [37].

Pharmacokinetic studies reveal significant first-pass metabolism of vitexin, with 94.1% degraded by intestinal β-glucosidases and only 5.2% metabolized in the liver. Despite low hepatic extraction, its oral bioavailability remains limited to 2.8% in rat models, primarily due to intestinal degradation [38]. This underscores the need for advanced delivery strategies, such as nanoparticle delivery systems or prodrug designs, to improve therapeutic delivery efficiency.

### 2.3. Biosynthesis and Production Strategies

In plants, vitexin is biosynthesized from apigenin using UDP-glucose or ADP-glucose as substrates in conjunction with the action of a C-glycosyltransferase enzyme, as documented in the studies of Fagopyrum esculentum M. cotyledons by Kerscher and Franz [39]. Currently, vitexin is mainly extracted from plants, involving ultrasonic extraction with alcoholic solvents followed by treatment with organic solvents and subsequent recrystallization or liquid-phase separation to isolate vitexin. However, the complex extraction process and low content of the target product limit its large-scale application.

The chemical synthesis of vitexin has been successfully performed in the laboratory using commercially available 2,4,6-trihydroxyacetophenone to form an O-glycoside intermediate, which is then converted via a Fries rearrangement into a C-glycoside derivative. This compound undergoes rearrangement and cyclization to produce a vitexin derivative, which is completely deprotected to yield vitexin [7]. However, the chemical synthesis of vitexin is limited by poor selectivity, low yield, and the need for multiple protection and deprotection steps.

Synthetic biology serves as an effective alternative method for the synthesis of flavonoids. Researchers coupled AcFNS with TcCGT and GmSUS to develop a one-pot enzymatic cascade for the biosynthesis of vitexin from (2S)-naringenin in vitro. This enzymatic cascade offers a widely applicable method for the structural modification of flavonoids that is both cost-effective and environmentally friendly [40]. Furthermore, a cost-effective coupling system for the synthesis of vitexin and orientin using TcCGT1 and GMSUS has been developed. In this system, the UDP–UDPG cycle and regeneration are conducted using inexpensive sucrose, which can effectively reduce the reaction cost and is more suitable for large-scale production [41]. The development of these methods not only provides new avenues for vitexin synthesis, but also opens new directions for the biomanufacturing of flavonoids.

## 3. Anticancer Effect of Vitexin

Emerging evidence highlights vitexin as a potent regulator of multiple cellular processes, with implications spanning cancer cell proliferation, cell cycle regulation, apoptosis, autophagy, metastasis, angiogenesis, epigenetic modifications, and tumor glycolysis inhibition. These effects have been documented across a wide range of cancers, including leukemia, oral cancer, hepatocellular carcinoma, esophageal cancer, colorectal carcinoma, glioblastoma, melanoma, nasopharyngeal carcinoma, lung cancer, renal cell carcinoma, breast cancer, cervical cancer, and others (Table 2). These findings collectively underscore vitexin’s potential as a multi-target therapeutic agent, capable of targeting the complex and heterogeneous characteristics of cancer.

**Figure 2:**
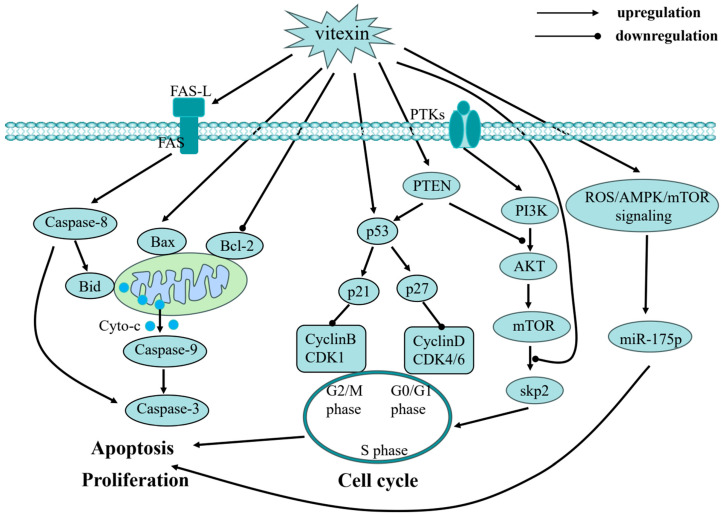
Cell cycle arrest, apoptosis induction and anti-proliferation of vitexin: vitexin exerts its anticancer effects through multiple molecular pathways. On one hand, it potently induces cancer cell apoptosis via activation of the Fas death receptor pathway, mitochondrial pathway, and p53 pathway. On the other hand, it effectively blocks cell cycle progression through upregulation of CDK inhibitors, downregulation of key cyclin/CDK complexes, and modulation of PTKs/PTEN and AMPK/mTOR signaling pathways. The synergistic action of these mechanisms ultimately achieves the anticancer effects of inhibiting cell proliferation and promoting cell death.

**Figure 3:**
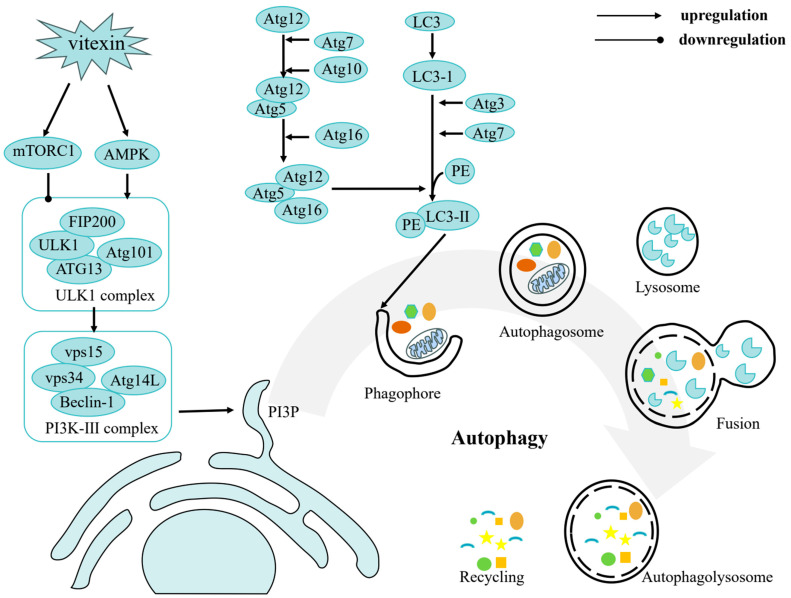
Autophagy of vitexin: vitexin induces autophagy by targeting the AMPK/mTORC1 signaling axis (activating AMPK while inhibiting mTORC1). This leads to ULK1 complex activation, subsequently triggering an ATG protein-mediated cascade (particularly the formation of the Atg12-Atg5-Atg16 complex and LC3-I to LC3-II lipidation), ultimately driving autophagosome formation. Following fusion with lysosomes, autophagosomes form autolysosomes for content degradation, with the breakdown products being recycled by the cell.

**Figure 4:**
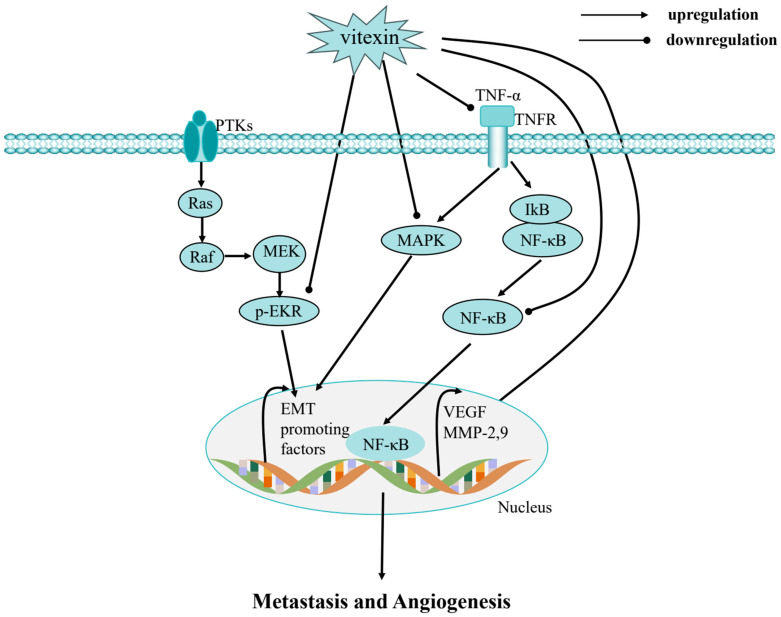
Metastasis and angiogenesis of vitexin: vitexin dually targets both PTKs and TNFR pathways, resulting in synergistic suppression of MAPK/ERK and NF-κB signaling cascades. This coordinated inhibition leads to significant downregulation of key pro-metastatic and angiogenic factors (including EMT markers, VEGF, and MMPs), ultimately blocking tumor metastatic potential and angiogenic capacity.

The molecular targets of vitexin are diverse, and its mechanisms of action are multifaceted. At the molecular level, vitexin exerts its effects primarily by modulating gene expression linked to key oncogenic processes. These include the regulation of pathways governing cancer cell proliferation, cell cycle progression, apoptosis, autophagy, metastasis, angiogenesis, and epigenetic modifications (Figure 2, Figure 3 and Figure 4). By targeting multiple nodes in these interconnected networks, vitexin offers a promising strategy for overcoming the challenges posed by cancer’s inherent complexity and resistance to conventional therapies.

### 3.1. Cell Cycle Arrest

The cell cycle, a tightly regulated process comprising the G1, S, G2, and M phases, relies on precise phase transitions to ensure controlled proliferation [70]. Dysregulation of this process is a hallmark of cancer, leading to uncontrolled cell division. Inducing cell cycle arrest represents a key therapeutic strategy to inhibit tumor growth and progression [71]. Vitexin has emerged as a promising therapeutic agent due to its ability to induce cell cycle arrest across multiple cancer types, offering a strategic approach to curb tumor growth and progression. This compound effectively modulates critical regulatory proteins to halt cell cycle progression at specific phases, thereby disrupting the uncontrolled proliferation characteristic of cancer.

Vitexin induces G0/G1 phase arrest through modulation of critical regulatory proteins. In hepatocellular carcinoma HepG2 cells, vitexin compound 1 promotes the expression of cyclin-dependent kinase inhibitors p21 and p27 while suppressing cyclin D1, thereby blocking cell cycle progression. This leads to a concentration-dependent inhibition of both anchorage-dependent and anchorage-independent growth [45]. Similarly, in nasopharyngeal carcinoma CNE1 and HK1 cells, vitexin downregulates cyclin D1 expression and upregulates p21 and p53, effectively arresting cells in G0/G1 [57].

Vitexin’s influence extends to the G1/S transition, where it exerts its effects by modulating key cyclins. In breast cancer cells, vitexin induces G1 phase arrest, which is attributed to the reduced expression of cyclins B1 and E, crucial drivers of the G1 to S phase transition [66]. This mechanism is further supported by studies in cervical cancer HeLa cells, where vitexin treatment elevates phosphorylated p53 levels while reducing cyclin B1 and E expression, thereby blocking the G1/S transition [68].

Additionally, vitexin demonstrates its therapeutic potential by targeting the G2/M transition. In glioblastoma cells, vitexin treatment significantly increases the proportion of cells in the G2/M phase while reducing those in the G1 phase [53]. In melanoma cells, 5 μM VB-1 induces G2/M phase arrest, while a higher concentration of 20 μM causes a more pronounced G2/M arrest and reduces the proportion of cells in the G0/G1 phase [56]. Furthermore, vitexin compound 1 has been shown to induce G2/M arrest in breast cancer cells via upregulation of p21 expression [62].

Overall, vitexin’s capacity to induce cell cycle arrest at critical transitions underscores its potential as a multi-faceted therapeutic agent. By targeting multiple regulatory proteins and cyclins, vitexin disrupts the progression of the cell cycle, offering a promising strategy for cancer treatment. Further exploration of these mechanisms in preclinical models is warranted to advance vitexin as a novel therapeutic option in oncology.

### 3.2. Apoptosis Induction

Apoptosis, or programmed cell death, is a critical process for suppressing tumor growth and is primarily initiated through caspase-dependent intrinsic or extrinsic pathways. This process involves a series of morphological changes, including membrane blebbing, nuclear fragmentation, cell shrinkage, chromosomal DNA fragmentation, and chromatin condensation [72]. Regulatory proteins such as the Bcl-2 family (pro-apoptotic Bax and anti-apoptotic Bcl-2) play essential roles in apoptosis and tumorigenesis [73]. Mitochondria are central to apoptosis, as the loss of mitochondrial membrane potential (MMP) can trigger the release of pro-apoptotic molecules [74]. Given its critical role in tumor suppression, apoptosis represents a promising anti-cancer strategy [75].

Vitexin has demonstrated significant potential in inducing apoptosis across various cancer types, making it a promising therapeutic candidate. In human leukemia cells (U937), vitexin reduces cell viability in a dose- and time-dependent manner by downregulating Bcl-2 and upregulating caspase-3 and caspase-9, thereby promoting apoptosis [42]. This mechanism is further supported by studies in esophageal cancer cells (EC-109), where vitexin upregulates p53 and downregulates Bcl-2, promoting apoptosis in a dose- and time-dependent manner [49]. Similarly, in oral cancer cells, vitexin treatment significantly upregulates p53 and downstream genes such as p21 and Bax [44].

Vitexin’s apoptotic effects are also linked to mitochondrial dysfunction and the activation of caspase pathways. In leukemia cells, vitexin induces apoptosis by damaging cell membranes, reducing MMP, increasing DNA fragmentation, and modulating apoptotic and survival proteins [45]. In human liver cancer SMMC-7721 cells, vitexin promotes apoptosis by upregulating p53, Bax, and caspase-3 [46]. In hepatocellular carcinoma cells, vitexin induces apoptosis in a concentration-dependent manner by increasing caspase-3 and cleaved caspase-3 expression while downregulating Bcl-2 [47]. Additionally, vitexin treatment increases levels of pro-apoptotic Bcl-2 family members (e.g., BAX and BID) and promotes caspase-3 cleavage, leading to apoptosis in colorectal cancer cells [51]. In lung cancer cells, vitexin reduces the Bcl-2/Bax ratio and promotes the release of cytochrome c from mitochondria to the cytosol, thereby increasing activated caspase-3 levels and inducing apoptosis [58].

Thus, vitexin’s ability to induce apoptosis across multiple cancer types highlights its potential as a multi-target therapeutic agent. Its effects on key proteins and pathways involved in apoptosis make it a promising candidate for further exploration in cancer treatment. Future research should focus on elucidating these mechanisms in greater detail to advance vitexin as a novel therapeutic option in oncology.

### 3.3. Autophagy Induction

Autophagy, a fundamental catabolic process, involves the transport of damaged cytoplasmic components and organelles to lysosomes for degradation, thus maintaining cellular energy homeostasis. This process has garnered significant attention for its critical role in tumor suppression [76]. Key regulators of autophagy include the mTOR, which as part of mTOR complex 1, governs both cell growth and autophagy [77]. Additionally, autophagy-related proteins such as ATG, Beclin1, and LC3 are essential for autophagosome formation and the execution of autophagy [78]. Notably, vitexin has been shown to induce autophagic cell death in various cancer cells, including colorectal carcinoma and hepatocellular carcinoma cells.

Research has demonstrated that vitexin treatment significantly decreases the expression of the autophagy-related protein LC3-II [47]. Studies have reported for the first time that vitexin effectively inhibits the proliferation of colorectal carcinoma cells by suppressing HSF-1 activity and inducing autophagic cell death through the activation of JNK and ApoL1 [50]. Furthermore, vitexin suppresses autophagosome formation by reducing the expression of autophagy marker proteins ATG5 and BECN1, and by inhibiting the conversion of LC3-I to LC3-II in HCT-116DR cells [51]. Conversely, vitexin has been shown to promote the expression of Beclin1, enhance the conversion of LC3-I to LC3-II, and increase the degradation of p62 in renal cancer cells (ACHN and OS-RC-2) [61]. In breast cancer cells, vitexin exposure results in elevated expression of ATG, Beclin1, and LC3-II genes [79]. Both LC3 and p62 serve as key regulatory proteins and markers of autophagy levels. In vivo studies in nude mice have demonstrated a significant increase in LC3-II expression and a decrease in p62 expression in tumor tissues from the vitexin treatment group compared to the control group, suggesting that vitexin may inhibit nasopharyngeal carcinoma growth by inducing both autophagy and apoptosis [80].

The multi-faceted effects of vitexin on autophagy highlight its potential as a therapeutic agent in cancer treatment. By modulating key autophagy-related proteins and pathways, vitexin offers a promising strategy for targeting cancer cells through both autophagy and apoptosis. Further investigation into these mechanisms is warranted to advance vitexin as a novel therapeutic option in oncology.

### 3.4. Anti-Proliferation

Inhibiting cell proliferation is critical for tumor suppression, as proliferation is a fundamental biological process and a key aspect of cellular differentiation [81]. Vitexin, an active component derived from Prosopis cineraria, has demonstrated significant anti-proliferative effects on chronic myeloid leukemia (K-562) cells. It decreases superoxide dismutase activity while increasing reactive oxygen species, nitric oxide, and malondialdehyde levels, inducing apoptosis in a dose- and time-dependent manner [82].

Similarly, vitexin suppresses the activation of the NF-κB signaling pathway and its key regulators (p65, IκBα, and IKKs) in nasopharyngeal carcinoma cells. This leads to apoptosis induction and inhibition of cell proliferation. Furthermore, in NPC xenograft mouse models, oral administration of vitexin at 30 mg/kg for two weeks reduces tumor growth by decreasing the expression levels of p-p65 and Cyclin D1 [57].

In renal cancer cells (ACHN and OS-RC-2), vitexin significantly inhibits cell growth and induces apoptosis and hyperautophagy in a dose-dependent manner. This effect is mediated by upregulation of the AMPK/mTOR and JNK pathways and downregulation of the PI3K/AKT/mTOR pathway [61]. Additionally, vitexin, in combination with syringate, inhibits the proliferation of breast cancer cells by targeting the GRP78/SREBP-1/SCD1 pathway [66].

### 3.5. Metastasis and Angiogenesis

Inhibiting metastasis and angiogenesis is crucial for cancer treatment, as they play significant roles in tumor progression and spread. Epithelial–mesenchymal transition (EMT) is a critical process driving cellular plasticity during development, and is believed to play a key role in cancer metastasis [83]. Several proteins and transcription factors, such as E-cadherin, Snail, and Twist, are known to drive the EMT process [84]. Matrix metalloproteinases (MMPs), particularly MMP2 and MMP9, are enzymes capable of degrading key components of the extracellular matrix, such as type IV and type I collagen. MMP9 is the largest enzyme in the MMP family, and MMP2 specializes in degrading type I collagen fibers. Both enzymes are closely associated with tumor migration and are often overexpressed in malignant tumors [85]. Additionally, angiogenesis, a process essential for both physiological and pathological events, is crucial for tumor metastasis. Vascular Endothelial Growth Factor (VEGF) binds to VEGFR to induce endothelial cell proliferation, and is closely related to tumor angiogenesis through promotion of new blood vessel formation [86]. Consequently, inhibiting angiogenesis is considered a viable strategy for cancer treatment [87].

Vitexin has demonstrated remarkable potential in suppressing these processes across various cancer types. For instance, vitexin has been shown to inhibit gastric cancer (GC) cell viability, migration, invasion, and EMT in a dose-dependent manner [65]. Studies have demonstrated that vitexin treatment effectively suppressed angiogenesis in endometrial cancer cells, as evidenced by reduced tube formation in vitro [63]. In oral cancer OC2 cells, vitexin induces the expression of plasminogen activator inhibitor-1 (PAI-1) and reduces the accumulation of active MMP-2 in a dose-dependent manner, leading to the inhibition of metastasis [44]. VB-1 also reduces VEGF secretion, thereby inhibiting endothelial tube formation [45]. Furthermore, vitexin downregulates the expression of MMP2 and MMP9, suppressing cancer cell migration and invasion [59]. By inhibiting the STAT3 signaling pathway, vitexin alters the expression of Arg-1, MR, and CD206 in M2-type macrophages, reducing the pro-metastatic capacity of the M2 phenotype in non-small cell lung cancer cells, ultimately exerting anti-lung cancer effects in vitro [60].

### 3.6. Epigenetic Modification

Epigenetic modifications of DNA and RNA play crucial roles in regulating growth, inheritance, and disease progression [88]. In recent years, the role of epigenetic modifications in the onset and progression of various malignant tumors has garnered significant attention. These modifications primarily involve DNA and protein alterations, such as methylation and acetylation [89]. Notably, vitexin has been shown to exert anticancer effects by regulating the expression of genes associated with epigenetic modifications in cancer cells.

Vitexin significantly inhibits the expression of HIF-1α, a tumor-associated oncogene regulated by the methylation of H3K27me3. Further analysis reveals that many of vitexin’s effects are mediated through histone modifications, particularly epigenetic changes. Key regulatory genes, including Bcl-2, P53, Caspases, and Bax, are influenced by methylation at various sites on histone H3, with H3K27me3 being especially relevant [90]. However, the potential for off-target effects must be considered. For example, flavonoids like vitexin may exhibit non-specific interactions with kinases such as PI3K or MAPK, which are critical in both cancer and normal cell signaling pathway s [91].

Additionally, a study has confirmed for the first time that vitexin modulates the expression profile of miRNAs in the MCF-7 breast cancer cell line. Treatment with vitexin significantly affects 20 miRNAs, notably upregulating let-7b and let-7c while downregulating miR-175p, thereby promoting apoptosis [67]. Notably, similar miRNA modulation (e.g., miR-175p downregulation) has been observed in non-cancerous cells exposed to high-dose vitexin (100 μM), highlighting the need for dose optimization to minimize off-tissue effects [92].

Vitexin’s ability to regulate epigenetic modifications highlights its potential as a therapeutic agent in cancer treatment. By targeting key genes and miRNAs involved in epigenetic regulation, vitexin offers a promising strategy for addressing the complex mechanisms underlying cancer progression. Further studies are needed to evaluate its specificity compared to established epigenetic drugs (e.g., HDAC inhibitors) and to identify biomarkers that predict selective on-target activity [93].

### 3.7. Inhibition of Tumor Glycolysis (Warburg Effect)

Emerging evidence demonstrates that vitexin effectively inhibits tumor cell metabolic reprogramming, particularly the characteristic Warburg effect—a phenomenon where cancer cells preferentially utilize glycolysis for energy production even under aerobic conditions. This metabolic shift, first described by Otto Warburg in 1924, provides proliferating tumor cells with essential biosynthetic precursors while creating an acidic microenvironment that promotes invasion and immune evasion. The heightened glycolytic flux in cancer cells is mediated through multiple mechanisms including upregulation of glucose transporters (GLUTs), rate-limiting glycolytic enzymes, and lactate dehydrogenase (LDHA), along with suppression of mitochondrial oxidative phosphorylation.

This flavonoid compound not only reduces glucose uptake in glioma cells by suppressing HIF-1α-mediated GLUT1 and GLUT3 expression [55], but also decreases lactate production in non-small cell lung cancer through PI3K/AKT/mTOR pathway-mediated inhibition of HK2 and LDHA activities [58]. Furthermore, in nasopharyngeal carcinoma cells, vitexin activates AMPK to shift cellular metabolism from glycolysis toward mitochondrial oxidative phosphorylation [80].

These findings establish vitexin as a potent modulator of tumor metabolism with significant potential for synergistic effects when combined with conventional metabolism-targeting therapies.

## 4. Synergistic Effects and Safety Considerations of Vitexin

Given its established anticancer potential, vitexin’s ability to modulate key signaling pathways and cellular processes presents promising opportunities for combination therapies. Research has demonstrated the potential of vitexin to synergize with both chemotherapy and radiation therapy, enhancing their therapeutic efficacy and reducing potential side effects.

For instance, research has shown that vitexin effectively potentiates the apoptosis-inducing activity of doxorubicin and sorafenib in hepatocellular carcinoma cells. By inhibiting key signaling pathways like PI3K/AKT and STAT3, vitexin disrupts tumor cell survival mechanisms and sensitizes them to the cytotoxic effects of these chemotherapeutics [48]. Another example is the investigation of vitexin’s impact on oxaliplatin sensitivity in colorectal cancer cells. Studies have demonstrated that vitexin can downregulate P-gp expression, a multidrug resistance protein, thereby reversing drug resistance and enhancing the effectiveness of oxaliplatin. Moreover, vitexin’s ability to induce apoptosis and arrest the cell cycle further enhances therapeutic response [94].

Beyond chemotherapy, vitexin’s role as a HIF-1α inhibitor offers intriguing possibilities for radiosensitization. HIF-1α plays a crucial role in tumor cell survival and adaptation to hypoxia, making its inhibition a valuable strategy for enhancing the effectiveness of radiation therapy. Research in nude mice with glioma demonstrated that vitexin, in combination with hyperbaric oxygen therapy, synergistically enhanced tumor cell sensitivity to radiation. This effect was attributed to vitexin’s ability to reduce antioxidant capacity in tumor tissues, leading to increased susceptibility to radiation-induced oxidative damage [55].

Furthermore, the novel combination of vitexin and aspirin has demonstrated synergistic effects against colorectal cancer, potentially by inhibiting NFKB1 activity. This leads to the suppression of COX-2 expression, ultimately reducing the proliferation of colorectal cancer cells. This finding highlights the potential of combining vitexin with other drugs that target specific pathways involved in cancer development and progression [95].

The targeted mechanisms of vitexin also open doors for further exploration in combination therapy. Its impact on epigenetic modifications and miRNA expression suggests potential synergies with other targeted drugs that modulate these pathways. For instance, combining vitexin with drugs that target histone methyltransferases or demethylases could offer a multi-faceted approach to cancer treatment, disrupting both tumor cell growth and survival mechanisms. Furthermore, vitexin’s anti-inflammatory and antioxidant properties could be beneficial in mitigating the side effects of chemotherapy and radiation therapy, such as nausea, vomiting, and skin irritation. Exploring these strategies may lead to innovative treatments that improve both patient outcomes and quality of life.

Notably, the therapeutic window of vitexin requires careful definition. While animal studies indicate low systemic toxicity, zebrafish embryo models reveal dose-dependent risks: concentrations ≥ 100 μM reduce survival rates, impair cardiac function, and increase ROS-mediated apoptosis [51]. Clinical translation must address its dual redox effects—exerting antioxidant protection at low doses but potentially triggering pro-oxidant toxicity at high doses or under specific microenvironments. Pharmacokinetic interactions are also critical, as in vitro studies confirm its inhibition of CYP3A4 and P-gp may alter the metabolism of drugs like paclitaxel, necessitating optimized dosing strategies in combination regimens. For sensitive populations, therapeutic drug monitoring is recommended, to guide personalized dosing.

## 5. Delivery Strategies for Vitexin in Cancer Therapy

Despite its promising anticancer properties, clinical application of vitexin faces several significant challenges, primarily due to its poor aqueous solubility, limited intestinal permeability, and extensive first-pass metabolism, which collectively result in low oral bioavailability [96]. Extensive research efforts have been dedicated to developing effective delivery systems that enhance the solubility, stability, and targeted delivery of vitexin. Nanotechnology offers a promising avenue for improving vitexin delivery [97]. Nanoparticles, due to their small size and large surface area, can significantly increase the solubility and bioavailability of hydrophobic compounds like vitexin [98]. Additionally, nanoparticles can be engineered to possess specific properties, such as targeted delivery to tumor cells, controlled release of the drug, and protection from degradation, further enhancing their therapeutic efficacy [99]. These strategies not only improve its pharmacokinetic profile, but also potentiate its therapeutic efficacy.

Several studies have successfully demonstrated the encapsulation of vitexin into various types of nanoparticles, resulting in improved solubility, stability, and bioavailability. For instance, researchers have explored the use of mung bean protein isolate (MBPI) nanoparticles as a delivery system for vitexin. Through ultrasound-assisted pH-shifting treatments, MBPI nanoparticles exhibit enhanced hydrophobic interactions with vitexin, leading to improved encapsulation efficiency and controlled release profiles [100].

Additionally, incorporating these MBPI nanoparticles into calcium carbonate microparticles further enhances their stability and reduces degradation during gastrointestinal digestion. Another approach involves the use of polymeric nanoparticles, such as poly (ethylene glycol) methyl ether-grafted chitosan (mPEG-g-CTS)/alginate (ALG) nanoparticles. These nanoparticles exhibit a spherical shape with a diameter ranging from 50 to 200 nm and a negatively charged surface, allowing for efficient encapsulation of vitexin. The mPEG-g-CTS/ALG nanoparticles demonstrate a high loading capacity and encapsulation efficiency, along with significant antioxidant activity [101].

Furthermore, the release of vitexin from these nanoparticles is pH-dependent, suggesting potential for targeted delivery to the intestinal tract. Furthermore, nanoemulsions have also emerged as a promising platform for vitexin delivery. Utilizing a mixture of Tween 80 and lecithin as emulsifiers, stable nanoemulsions with small oil droplets can be prepared, effectively encapsulating vitexin with high efficiency. These nanoemulsions exhibit enhanced stability during storage and protect vitexin from degradation during in vitro gastrointestinal digestion [102].

These examples illustrate the potential of nanotechnology in improving the delivery and bioavailability of vitexin, paving the way for its development as a novel anticancer therapeutic. Further research and development efforts are needed to optimize these delivery systems and investigate their efficacy in vivo, ultimately leading to the translation of vitexin-based therapies for the treatment of cancer.

## 6. Future Perspectives

Vitexin has emerged as a promising candidate in cancer therapy, offering a multi-faceted approach to target the complex and heterogeneous nature of cancer. However, several critical areas require further investigation to fully realize its therapeutic potential.

Despite significant progress, the precise mechanisms underlying vitexin’s effects on cancer cells require deeper investigation. To establish causal relationships, CRISPR-Cas9-mediated gene editing should be prioritized. For instance, isogenic cell lines with STAT3 knockout or constitutive activation can clarify whether vitexin’s anticancer effects are STAT3-dependent. Parallel genome-wide CRISPR screens may systematically map synthetic lethal interactions between vitexin and cancer driver genes, revealing context-specific mechanisms. Advanced techniques such as single-cell RNA sequencing coupled with spatial transcriptomics should be employed to identify specific molecular targets and pathways, which will enhance our understanding of how vitexin interacts with cancer-specific biomarkers and tumor microenvironments. Additionally, identifying predictive biomarkers for vitexin’s efficacy is essential. Research should focus on discovering biomarkers that can stratify patients who are most likely to benefit from vitexin-based therapies, thereby personalizing treatment approaches. Vitexin’s ability to modulate multiple signaling pathways makes it an ideal candidate for combination therapies. Preclinical studies should explore synergistic combinations with immunotherapies, targeted therapies, and emerging treatments such as photothermal therapy and sonodynamic therapy. These combinations may overcome resistance mechanisms and enhance therapeutic outcomes.

Another primary issue is its poor absorption and low bioavailability due to extensive first-pass metabolism, primarily in the intestine. This significantly reduces its therapeutic potential. While nanotechnology offers promising avenues for improving vitexin’s delivery, further optimization is needed. Future studies should aim to develop smart nanocarriers capable of responding to tumor-specific stimuli for controlled drug release. Additionally, the long-term safety and biocompatibility of these nanosystems must be rigorously evaluated. Bridging the gap between preclinical success and clinical application remains a challenge. Well-designed phase I/II clinical trials are needed to assess vitexin’s safety, pharmacokinetics, and efficacy in humans. Adaptive trial designs and real-world evidence generation could accelerate its translation into clinical practice.

Furthermore, understanding how cancer cells develop resistance to vitexin is crucial. Studies should investigate the genetic and epigenetic changes that drive resistance, informing strategies to mitigate these effects and sustain treatment responses. Given the global nature of cancer research, international collaborations across institutions and countries can expedite vitexin’s development. Shared databases, standardized protocols, and joint funding initiatives will facilitate knowledge exchange and resource optimization. In conclusion, vitexin holds significant promise for cancer treatment, but its successful translation into clinical practice requires addressing these challenges through innovative research and collaborative efforts. By focusing on these areas, vitexin could evolve into a cornerstone of next-generation cancer therapies.

## 7. Conclusions

Vitexin, a natural flavonoid found in various plants, has shown significant potential as an anticancer agent, through its diverse biological activities and effects on multiple cellular processes. This review highlights vitexin’s ability to regulate cell cycle progression, induce apoptosis, modulate autophagy, inhibit metastasis and angiogenesis, and influence epigenetic modifications, demonstrating its multi-target therapeutic potential. Although preclinical studies have revealed promising anticancer effects, it is important to acknowledge that current evidence predominantly relies on in vitro and animal models. These experimental systems, while invaluable for mechanistic exploration, cannot fully recapitulate the complexity of the human tumor microenvironment and interindividual variability. The pharmacokinetic profiles and long-term safety of vitexin in human subjects remain to be systematically investigated. Further research is needed to fully understand its mechanisms, optimize delivery systems, and evaluate its safety and efficacy in clinical settings. Vitexin’s potential for combination therapies and its favorable safety profile make it a compelling candidate for the development of novel cancer treatments.

## Figures and Tables

**Table 1:** Plant Sources of Vitexin.

No.	Plant Species (Latin Name)	Part Used	Reference
1	*Mimosa diplotricha* Sauvalle	aerial parts	[23]
2	*Senna siamea*	leaves and bark	[24]
3	*Garcinia mckeaniana*	leaves	[25]
4	*Hypericum coadunatum* Sm. ex Link Buch.	aerial parts	[26]
5	*Humulus japonicus*	aerial parts	[27]
6	*Polygonum orientale*	whole plants	[28]
7	*Passiflora spp.*	fruits	[29]
8	*Vigna radiata*	seeds	[30]
9	*Fagopyrum esculentum*	seeds	[31]
10	*Anagallidium dichotomum* (L.) Griseb	flowers, leaves, stems	[32]
11	*Trema orientalis* L.	ripe fruits	[33]
12	*Clinacanthus nutans*	leaves	[34]

**Table 2:** The underlying mechanisms of vitexin against different cancers.

Cancer	Models (In Vitro/In Vivo)	Biological Activities	Molecular Mechanisms	IC50 (μM)	Incubation Time	References
Leukemia	U937 cells (in vitro)	cytotoxicity and apoptosis induction	caspase-3, -7 and caspase-9 activities↑ Bcl-2↓	22.5	24	[42]
	K-562 cells (in vitro)	apoptosis induction	MMP↓ ROS↑ RAS↓ RAF↓ p38↑ BCL-2↓ procaspase-9↓ pro-caspase-3↓ BAX↑	65.7	48	[43]
Oral cancer	OC2 cells (in vitro)	induce apoptosis, inhibit proliferation and metastasis	caspase-3↑ p53↑ p21↑ Bax↑ PCNA↓ PAI-1↑ MMP-2↓	40	24	[44]
Hepatocellular carcinoma	Hep3B, Huh-7, HepG2 and L-02 cells (in vitro)	induce G1/G0 phase arrest, inhibit growth and angiogenesis	P-PI3K↓ p-Akt↓ FOXO3a↑ p-FOXO3a↓ p21↑ p27↑ cyclin D1↓ VEGF↓	50	48	[45]
	SMMC-7721 cells (in vitro)	inhibit proliferation and induce apoptosis	Bcl-2↓ Casepase-3↑ Bax↑ P53↑ PARP↑	32.7	48	[46]
	SK-Hep1 and Hepa1-6 cells (in vitro)	apoptosis induction and autophagy suppression, exert an inhibitory effect on HCC tumor growth	Caspase-3↑ Cleave Caspase-3↑ Bcl-2↓ LC3 II↓ p-JNK↑ p-Erk1/2↓ Ki67↓ MMP-2↓	35	48	[47]
	HepG2, Hep3B, HCCLM3, and PLC/PRF5 cells (in vitro)	mitigate the survival and invasion of HCC cells	p-STAT3↓ cyclin D1↓VEGF↓ Bcl-2↓ Bcl-xL↓ Mcl-1↓ survivin↓ cleavage of procaspase-8 and procaspase-3↑	52.1	24	[48]
Esophageal cancer	EC-109 cells (in vitro)	inhibit cell growth and induce apoptosis	p53↑ bcl-2↓	35	24	[49]
Colorectal carcinoma	HCT-116 cells (in vitro), xenograft model (in vivo)	autophagy induction, inhibit the cell growth	HSF-1↓ JNK↑ PI3K↓p-Akt↓ p-mTOR↓ p62↓ Bcl-2↓ Beclin-1↑Atg5↑ LC3-II↑ p-JNK↑ LC3-II↑ ApoL1↑	25	48	[50]
	HCT-116DR cells (in vitro), xenograft model (in vivo)	induce apoptosis through suppression of autophagy	ROS↑ BID↑ Bax↑ cytochrome c↑ ATG5↓ Beclin-1↓ LC3-II↓	55	72	[51]
	HCT-116WT, HCT-116, p53-KO,HCT-116 PUMA-KO, HCT-116, BAX-KO and LoVo cells (in vitro), xenograft mouse model (in vivo)	suppress proliferation and induce apoptosis	p53↑ PUMA↑ Bax↑	20	48	[52]
Glioblastoma	LN-18 cells (in vitro)	induce G2/M cell cycle arrest and cell apoptosis	Akt/mTOR↓ cleaved-PARP↑ p-Akt↓ p-mTOR↓	30	48	[53]
	U251 cells (in vitro)	inhibit proliferation and invasion, induce apoptosis	JAK/STAT3↓	40	48	[54]
Glioma	SU3 cells (in vitro), BALB/c nude mice (in vivo)	cooperate with HBO to sensitize the glioma radiotherapy	HIF-1α↓ VGEF↓ GLUT-1↓ GLUT-3↓	29	48	[55]
Melanoma	A375, Sk-Mel-5 and Sk-Mel-28 vemurafenib-resistant A375 cells (in vitro)	DNA damage, G2/M cell cycle arrest and apoptosis	ROS↑ P21↑ PUMA↑GADD45A↑ MCM6↓CDK1↓ CDK6↓ CYCE↓ CYCA↓	26	48	[56]
Nasopharyngeal carcinoma	NPC cells CNE1, CNE2, HK1 and HNE1 cells (in vitro)	induce G0/G1 cell cycle arrest and apoptosis, inhibit NF-κB signaling	Cyclin D1↓ p21and p53↑ cleaved PARP↑ Bcl-2 and Mcl1↓ IKK↓ NF-κB↓	24	48	[57]
Lung cancer	A549 and 16HBE cells (in vitro)	induce apoptosis and inactivate PI3K/Akt/mTOR signaling	Bcl-2↓ Bax↑ cleaved caspase-3↑ MMP↓ cytochrome c↑ p-PI3K, p-Akt and p-mTOR↓	28	48	[58]
	A549 cells (in vitro)	induce apoptosis, inhibit migration and invasion	caspase3, caspase9, Bcl-2 and bax↑ MMP2 and MMP9↓	27	48	[59]
	RAW264.7 and A549 cells (in vitro)	decrease migration	iNOS, IL-1β, Arg-1,MR and p-STAT3↓	N/A	N/A	[60]
Renal cell carcinoma	OS-RC-2 and ACHN, HK-2 cells (in vitro)	induce apoptosis and hyperautophagy, up-regulate AMPK/mTOR and JNK pathways, down-regulate PI3K/Akt/mTOR pathways	caspase-3, caspase-9, cleaved caspase-3, and cleaved caspase-9↑Beclin1 and LC3↑ p62↓ p-AMPK↑ p-JNK↑ P-PI3K and p-AKT↓	25	48	[61]
Ovarian cancer	HO8910 and SKOV3 cells (in vitro), xenograft tumor model (in vivo)	induce apoptosis and G2/M arrest	caspase-3↓ cleaved caspase-3↑ p21↑	26	48	[62]
Endometrial cancer	HESCs, HEC-1B and Ishikawa cells (in vitro)	suppress the proliferation, angiogenesis, stemness and the PI3K/AKT pathway	Ki-67 and PCNA↓ VEGFA and FGF2↓ OCT4 and Nanog↓ P-PI3K and p-AKT↓	24	48	[63]
Gastric cancer	AGS, CRL-1739, GES-1, SGC-7901cells (in vitro)	induce autophagy and apoptosis	p-PI3K, p-AKT and p-mTOR↓	25	48	[64]
	AGS, CRL-1739, GES-1, SGC-7901cells (in vitro)	suppress the migration, invasion, and EMT, inhibit the activation of PI3K/AKT/mTOR/HIF-1α pathway	cadherin↑ N-cadherin, MMP9 and MMP2 ↓HMGB1, p-PI3K,p-AKT, p-mTOR andHIF-1α↓ Ki67↓	25	48	[65]
Breast cancer	MDA-MB-231 and MCF-7 cells (in vitro)	inhibit proliferation	ki-67↓ SCD1↓ SFA↑ LDs↑	30	48	[66]
	MCF-7 cells (in vitro)	induce apoptosis	regulation of specific miRNAs expression	32	48	[67]
Cervical cancer	HeLa cells (in vitro)	induce apoptosis and phase arrest	Bcl-2↓ Bax, caspase-3↑ p-P53↑ cyclin B1 and cyclin E↓	22	48	[68]
	Hela and Siha cells (in vitro)	reduce cell proliferation, migration, invasion and angiogenesis	VEGFA/VEGFR2↓	23	48	[69]

Note: ↑: Increase; ↓: Decrease; N/A: Not available. MMP: Mitochondrial Membrane Potential; ROS: Reactive Oxygen Species; RAS: Rat Sarcoma; RAF: Rapidly Accelerated Fibrosarcoma; BCL-2: B-cell lymphoma 2; BAX: BCL-2-associated X protein; PCNA: Proliferating Cell Nuclear Antigen; PAI-1: Plasminogen Activator Inhibitor-1; VEGF: Vascular Endothelial Growth Factor; PARP: Poly (ADP-ribose) Polymerase; LC3-II: Microtubule-associated protein 1A/1B-light chain 3; JNK: c-Jun N-terminal Kinase; Erk1/2: Extracellular signal-regulated kinase 1/2; STAT3: Signal Transducer and Activator of Transcription 3; HIF-1α: Hypoxia-Inducible Factor 1-alpha; GLUT-1/3: Glucose Transporter 1/3; GADD45A: Growth Arrest and DNA Damage-inducible 45 Alpha; MCM6: Minichromosome Maintenance Complex Component 6; CDK1/6: Cyclin-Dependent Kinase 1/6; CYCE/A: Cyclin E/A; NF-κB: Nuclear Factor Kappa B; IKK: IκB Kinase; iNOS: Inducible Nitric Oxide Synthase; IL-1β: Interleukin-1 Beta; Arg-1: Arginase-1; MR: Mannose Receptor; AMPK: AMP-activated Protein Kinase; mTOR: Mammalian Target of Rapamycin; EMT: Epithelial-Mesenchymal Transition; HMGB1: High Mobility Group Box 1; SCD1: Stearoyl-CoA Desaturase-1; SFA: Saturated Fatty Acids; LDs: Lipid Droplets.
